# Preparation and Characterization of Bigels from Psyllium Husk Seed Hydrogel–Beeswax Oleogel: As a Fat Replacer in Cakes

**DOI:** 10.1021/acsomega.5c03403

**Published:** 2025-07-23

**Authors:** Alican Akcicek, Fatma Gultekin, Salih Karasu

**Affiliations:** † Faculty of Tourism Department of Gastronomy and Culinary Arts, 52980 Kocaeli University, Kartepe, Kocaeli 41080, Turkey; ‡ Faculty of Tourism Department of Gastronomy and Culinary Arts, Kocaeli University, Kartepe, Kocaeli 41080, Turkey; § Faculty of Arts and Social Sciences Department of Gastronomy and Culinary Arts, Istanbul Galata University, Beyoglu, Istanbul 34430, Turkey; ∥ Department of Food Engineering, Faculty of Chemical and Metallurgical Engineering, Yildiz Technical University, Davutpasa Campus, 34210 Istanbul, Turkey

## Abstract

This study used PHP (psyllium husk seed powder) and BW (beeswax) to create a hydrogel and Oleogel for bigel production. The resulting bigels were utilized as a fat replacement in cake composition. Bigel manufacturing employed 5 and 10% PHP hydrogels and 7.5% BW Oleogel at various HG/OG ratios. Visual appearance results indicated that gelation was verified by inverting the samples in plastic tubes, which exhibited no flow under gravity. Also, the samples became bright yellow as the Oleogel proportion increased. Microscopy revealed that the bigel with a hydrogel/Oleogel volume ratio of 75:25 formed bicontinuous structures. Fourier transform infrared spectroscopy (FTIR) spectra of the Oleogel, hydrogels, and bigels showed no new peaks identified in the bigel samples with different fractions of Oleogel. The bigel samples showed that the *G*′ values were higher than the *G*″ values, indicating that the samples showed viscoelastic solid behavior. An increase in the Oleogel ratio in bigel samples led to improved viscoelasticity. The hydrogel ratio exhibited the highest thermal stability and a greater *G*′ value than the Oleogel, particularly at 5%-75 PHP and 10%-75 PHP across different temperatures. In addition, the hardness and chewiness values of bigel samples increased with hydrogel concentrations. The bigel stability result showed that the 10%-25 PHP had the lowest accelerated percolation rate, suggesting it was the most stable sample and retained more liquid. Cake samples created with palm oil, 5%-25 PHP, and 10%-25 PHP had a crumb structure with fine air cells evenly distributed. 10%-25 PHP and 5%-25 PHP cake samples showed the highest hardness values and no significant change with the control palm cakes (p 0.05). Furthermore, based on the Δ*E* value, 5%-50 PHP and 10%-25 PHP cakes had the least color change in the crust and the crumb of the cake. The 10%-25 PHP cake samples would be a better choice as they could be considered an alternative to palm oil in cake in terms of visual appearance, textural properties, and color properties.

## Graphical abstract

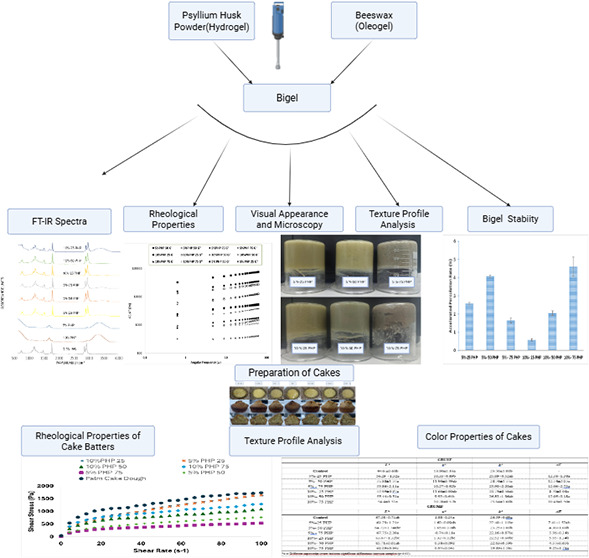

## 1 Introduction

Solid fats, such as butter and hydrogenated vegetable oils, have been widely used in baked goods since the modern food industry’s inception due to their capacity to give desirable textures, flavors, and tastes. Their high saturated fatty acid (SFA) content makes them prone to causing chronic food-related disorders such as obesity, atherosclerosis, and diabetes mellitus.^1–3^ As a result, the food industry has difficulty developing alternatives to solid fats that may minimize or replace saturated and trans fats while preserving product textural and nutritional quality.^1,4^

Gel-based fat substitutes such as Oleogel and emulgel are promising for physically encasing liquid oils in a three-dimensional (3D) gel-like structure while preserving the texture and improving the nutritional value of baked goods. Bigels made by mechanically combining hydrogel and Oleogel under particular temperatures and conditions are examples of novel fat replacement techniques in this respect.^5–7^ They combine the benefits of two gel phases while providing better characteristics.^8^ As opposed to emulgels, which only gel one phase, bigels exhibit greater stability and sensory quality because they have two gelled phases.^5,6,9^ Oleogel-in-hydrogel (O/W), hydrogel-in-Oleogel (W/O), or bicontinuous phases might be present depending on the polarity of the continuous phase. Bigel’s structural and physicochemical characteristics are influenced by the ratio of Oleogel to hydrogel, the materials used for both, shearing speed, and gel particle size.^10,11^

Bigel, a novel fat replacement in the food industry, has attracted attention for its potential to mimic high-fat meals’ sensory and textural features while providing a more nutritional profile than standard fats.^12,13^ Bigels are helpful as a solid fat alternative in baked products since they are both inexpensive and convenient. Because of their great diversity and distinct solid-like qualities without any chemical bonding, they can be used as a suitable fat substitute in baked goods that have functions comparable to those of commercial solid fats.^6^

The application of bigels as fat replacers were reported by ref 14 for low-fat burger,^15^ for cookies,^1^ for bread,^13^ for low-fat mayonnaise,^16^ for shortbread cookies,^17^ for whipped cream,^18^ and for fermented sausages.

PHP is made from the husk of psyllium seeds, which dissolve in water to create a viscous colloidal solution.^19,20^ PHP contains over 80% dietary fiber, and the polymer complex arabinoxylan, abundant in PHP, has 1,4-β-D-xylopyranose as its primary chain, with arabinose at the end and side chains.^20^ PHP has special functional qualities due to its polysaccharide, including a very high water binding capacity, a thickening agent, a significant increase in viscosity, and a superior gel-forming ability.^21,22^ PHP is unique among polysaccharide colloidal compounds due to its low cost, ease of production, and stability. PHP also has outstanding gelation, thickening, and water-absorbent qualities.^23^ PHP which is made from the husk of psyllium seeds from Plantago ovata, has garnered a lot of interest lately because of its many health advantages, rich dietary fiber content (81%), high soluble dietary fiber (70%), and clean label characteristics.^20,21,24^ Psyllium has been employed as a binding, emulsifying, gelling, suspending, and stabilizing agent because of its hydrophilicity, which makes it an ideal candidate for these functions.^25^ PHP can satisfy customers’ desire for a lower intake of saturated fatty acids by acting as a dietary fiber supplement and a fat substitute.^23^

Despite the potential uses of bigels as a fat substitute in recent years, studies in this subject are limited due to their remarkable adaptability. However, similar studies in the development of bigels but using other ingredients were in the literature for the preparation of hydrogels and oleogels such as guar gum and rice bran wax,^3^ starch and monoglyceride,^26^ hydroxypropyl methyl cellulose and beeswax,^1^ and soy lecithin–beeswax and flax seed gum.^27^ Despite several researches in the literature, the assessment of the hydrogel/Oleogel (HG/OG) ratio in relation to hydrogelator concentrations needs further investigation prior to its application in food. For this aim, bigels were created by combining the Oleogel formed with BW and the hydrogel prepared with PHP in various ratios, and their texture, rheological characteristics, and microstructures were studied in this study. Furthermore, the best bigels were selected, and their effect on the qualitative features of cakes as a palm oil alternative was studied. Thus, the goal is to promote the use of PHP bigels as a solid fat alternative in food products.

## 2 Materials and Methods

### 2.1 Materials

In this study, extra virgin olive oil used as the oil phase, psyllium husk powder, beeswax palm oil, wheat flour, sucrose, baking powder, and homogenized egg were obtained from local markets in Turkey. All chemical materials and standards were purchased from Sigma Chem. Co. (St. Louis) and Merck (Darmstadt, Germany).

### 2.2 Methods

#### 2.2.1 Preparation of Bigels

The bigel samples were fabricated using the modified method.^28^ The hot homogenization procedure was employed in the manufacturing of bigels. This method makes it easier for the Oleogel and hydrogel phases to integrate and disperse, resulting in the creation of stable bigel structures that could be used in food products.^29^

PHP powder was dissolved in distilled water at 5 and 10% (w/w). Then, the hydrogel samples were prepared in a magnetic stirrer at 90 °C. Also, the Oleogel was fabricated by dissolving 7.5% (w/w) beeswax in olive oil at the same temperature at 300 rpm until fully melted. Different hydrogel and Oleogel ratios were created to form bigel samples (25%:75%, 50%:50%, and 75%:25%). The hydrogel and Oleogel samples were heated and homogenized using an ultraturrax at 10,000 rpm for 3 min. Then, the bigel samples were cooled to room temperature and kept in the refrigerator for 1 day. The samples were stored at 4 °C until the analysis. The formulation of Oleogel, hydrogels, and bigels are presented in Table 1.

**Table 1:** Formulations of Oleogel, Hydrogel, and Bigel Samples

sample	hydrogel/oleogel ratio
7.5% BW	0:100
5% PHP	100:0
10% PHP	100:0
5%-25 PHP	25:75
5%- 50 PHP	50:50
5% - 75 PHP	75:25
10%- 25 PHP	25:75
10% - 50 PHP	50:50
10% - 75 PHP	75:25

#### 2.2.2 FTIR

The stability and compatibility of the gels were determined by using a Bruker Tensor 27 FTIR spectrometer (Bremen, Germany) equipped with an ATR accessory with a diamond crystal module. The detector was a DLaTGS with a KBr beam splitter. FTIR spectra of HG, OG, and BG samples were acquired from 3800 to 600 cm^–1^ at 4 cm^–1^ resolution, accumulating 64 scans per spectra.^30^ The composition of the gel sample was verified by using the OPUS program, version 7.2 for Windows from Bruker GmbH.

#### 2.2.3 Rheological Properties

A rheometer (MCR 302; Anton Paar, Austria) associated with a Peltier heating system that was controlled by the temperature, and stress was used to assess the rheological properties of the HGs, OGs, and BGs samples. A PP50 rheometer probe was put into the parallel plate with a 0.5 mm gap between the sample plates. The rheological analysis was carried out three times at 25 °C, except for the temperature sweep analysis.

A parallel plate setup was used to perform a dynamic rheological study of HGs, OGs, and BGs samples.

An amplitude sweep test was initially used to identify the linear viscoelastic region (LVR) with a strain value of 0.1%. LVR was subjected to a frequency sweep test at 0.1–10 Hz and 0.1–64 (ω) angular velocity. The angular velocities of the samples were used to determine the storage modulus (*G*′) and loss modulus (*G*″). The dynamic rheological parameters were determined using a power law model and nonlinear regression^31^

1 $$G^{\prime }=K^{\prime }{\left(\omega \right)}^{n\prime }$$

2 $$G^{\prime \prime }=K^{\prime \prime }{\left(\omega \right)}^{n\prime \prime }$$

The formula uses the following values to represent: *G′* is the storage modulus′*G*′′ is the loss modulus″, ω is theangular velocity value (rad s^–1^), *K*′ and *K*″ are consistency index values, and *n*′ and *n*″ are flow behavior index values.

The temperature-dependent properties of the bigel, hydrogel, and Oleogel samples were examined by using a temperature sweep test. G′ values of the samples were assessed at temperatures between 20 and 80 °C, with a 5 °C rise in temperature for 1 min.

#### 2.2.4 Visual Appearance and Microscopy

A smartphone (Redmi Note 8 Pro, China) was used to take images of the BGs, cake dough, and cake samples. A polarized light microscope with a digital camera was used to examine the materials. A little bigel was put on a glass slide for a microscope and covered with a coverslip. A 40× magnification was used to take images of the samples (Olympus BX41, Tokyo, Japan).

#### 2.2.5 Texture Profile Analysis of Gels

A texture analyzer (TA.XT2 Plus, Godalming, U.K.) was used to determine the hardness, cohesiveness, gumminess, chewiness, and springiness of the OGs, HGs, and BGs. A needle probe P/5 (5 mm in diameter) was used to conduct a penetration test on the samples. The trigger force was 5 g, the test speed was 5 mm/s, the pretest speed was 1 mm/s, and the post-test speed was 5 mm/s.

#### 2.2.6 Bigel Stability

The bigel’s stability was described by the liquid precipitating following centrifugation.^17,32^ Following a 24 h cooling period, the bigels (5–6 g) were transferred to preweighed 50 mL centrifuge tubes. The tubes were then centrifuged for 10 min at 5000 rpm. The mass of the solids in the centrifuge tube was measured after the discharged liquid was removed. The bigel’s stability was indicated by its “accelerated precolation rate” (APR), which was determined using the following formula

$$\mathrm{APR}=\frac{\mathrm{Wb}-\mathrm{Wa}}{\mathrm{Wb}}\times 100\%$$

where Wa represents the mass of the gel following centrifugation and the removal of the exudate and Wb is the mass of the gel sample added before centrifugation.

#### 2.2.7 Preparation of Cakes

The Cakes were produced with minor modifications based on the study. Cake samples were made using 36% wheat flour, 20% sucrose, 16% oil phase (palm oil or bigels), 2% baking powder, and 26% homogenized egg. The ingredients were combined until a homogeneous cake batter was created. Cake batter samples were baked in an oven at 170 °C for 30 min.

#### 2.2.8 Rheological Properties of Cake Batters

The flow behavior in the rheological characteristics of the cake batter samples was investigated in the range of 0–100 shear rate (s^–1^). Shear stress and apparent viscosity values related to the shear rate were measured. The power law model and nonlinear regression were used to calculate the flow behavior and rheological characteristics.

3 τ=K×γn

The formula specifies τ as the shear stress (Pa), *K* as the consistency index (Pa.s^n^), γ as the shear rate (s ^–1^), and *n* as the flow behavior index.

#### 2.2.9 Textural Properties of the Cakes

The texture profile analysis of cakes was measured by modifying the method used by Liu et al.^33^ The TA-XT Plus texture analyzer (Stable Microsystems, Godalming, U.K.) was used to measure the texture at 20 °C. A P/36 R cylindrical probe was used for the analysis. The test speed was 5 mm/s, the pretest speed was 1 mm/s, and the post-test speed was 5 mm/s, with a 5 g trigger force.

#### 2.2.10 Color Properties of the Cakes

A colorimeter (Konica Minolta CR-400, NJ) was used to measure the color parameters L*, a*, and b*. The color analysis assessed the brightness (L*), redness (a*), and yellowness (b*) after the device was calibrated at room temperature and using a white ceramic plate. The color difference (ΔE*) between the bigels and palm oil-made cakes was measured^34,35^

$$\Delta E=\sqrt{{\left(L_{0}^{*}-L^{*}\right)}^{2}+{\left(a_{0}^{*}-a^{*}\right)}^{2}+{\left(b_{0}^{*}-b^{*}\right)}^{2}}$$

The colorimetric characteristics of palm oil cakes are *L*_0_*, *a*_0_*, and *b*_0_*, whereas those of bigel-made cakes are L*, a*, and b*.

#### 2.2.11 Statistical Analysis

Every measurement and analysis was carried out three times. **The results were given as mean and standard deviation**. The JMP 18 package software was used to conduct statistical analysis on the data collected at the end of the study. Analysis of variance (ANOVA) (*p* < 0.05) and the Tukey–Kramer HSD comparison test were used to determine the difference between group means. The parameters of the power law model were determined using nonlinear regression analysis with STATISTICA software (StatSoft Inc. in Tulsa, U.K.).

## 3 Results and Discussion

### 3.1 Visual Appearance and Microstructure Properties

Figure 1 displays the visual appearance of bigels in varying Oleogel/hydrogel ratios. Hydrophilic and oleophilic gelators must have the appropriate intrinsic molecular properties to create bigel.^36^

**Figure 1:**
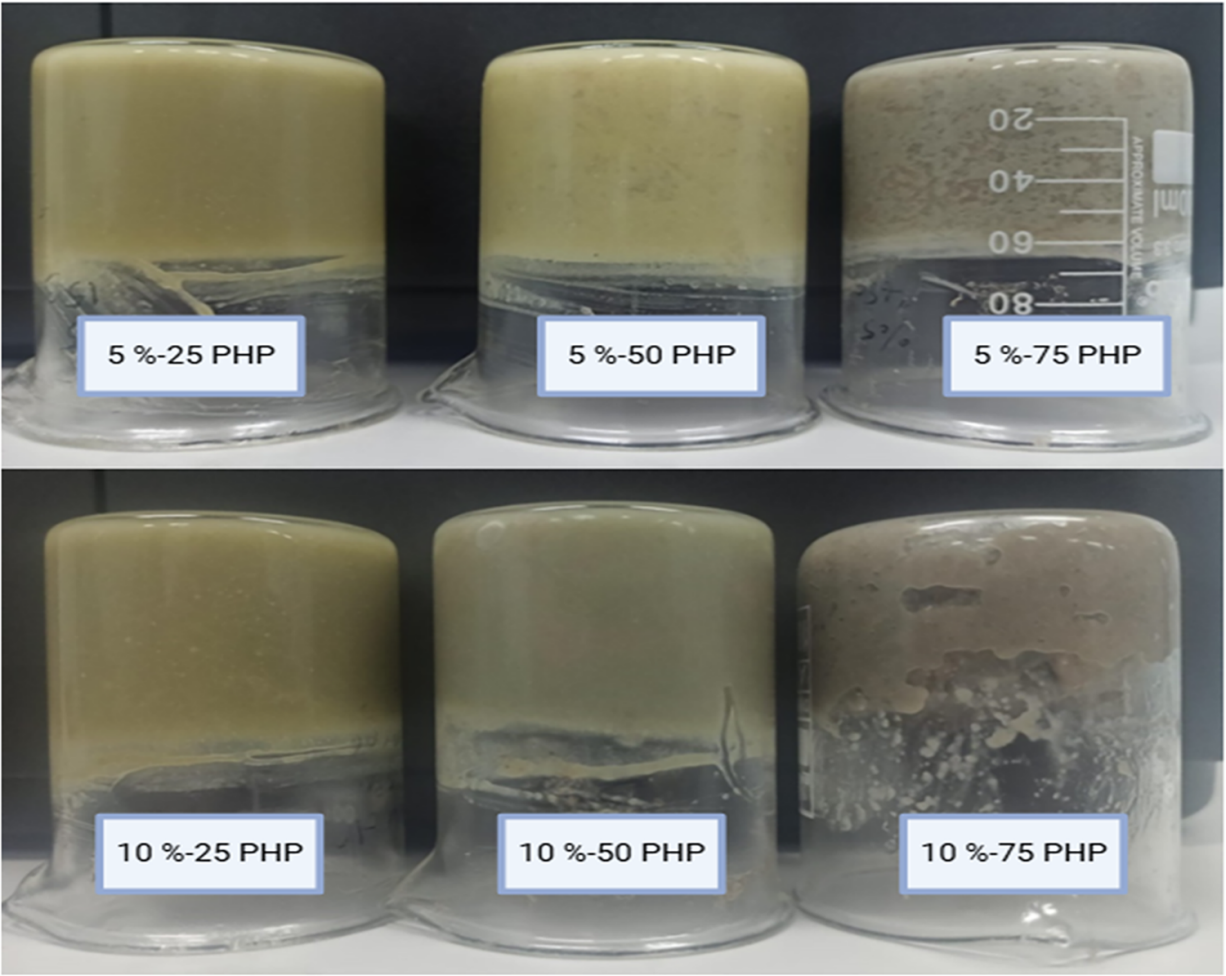
Visual appearance of the bigels (5%-25 PHP: containing 5% psyllium husk seed powder with 25:75 psyllium husk seed powder hydrogel to beeswax Oleogel ratio, 5%-50 PHP: containing 5% psyllium husk seed powder with 50:50 psyllium husk seed powder hydrogel to beeswax Oleogel ratio, 5%-75 PHP: containing 5% psyllium husk seed powder with 75:25 psyllium husk seed powder hydrogel to beeswax Oleogel ratio, 10%-25 PHP: containing 10% psyllium husk seed powder with 25:75 psyllium husk seed powder hydrogel to beeswax Oleogel ratio, 10%-50 PHP: containing 10% psyllium husk seed powder with 50:50 psyllium husk seed powder hydrogel to beeswax Oleogel ratio, 10%-75 PHP: containing 10% psyllium husk seed powder with 75:25 psyllium husk seed powder hydrogel to beeswax Oleogel ratio).

Bigel’s self-standing capacity was examined using the tube inversion method, which enabled the identification of the phase separation phenomena.^28^

Gelation was verified by inverting the samples into plastic tubes, which exhibited no flow under gravity. The samples became bright yellow as the Oleogel proportion increased. The color of the samples was created by light diffraction from scattered droplets or the oil–water contact.^37^ Each sample featured a smooth surface and was firm to the touch. Similar results were reported for the k-carragenan hydrogel and monoglyceride oleogels based on bigels.^38^

Figure 2 displays the bigels’ microstructural characteristics. Depending on the characteristics of the gel and the manufacturing process, bigels can be organized as water-in-oil, oil-in-water, or bicontinuous emulsion systems.^36^ Hydrogel-in-Oleogel microstructure was seen in the bigel samples with 25% H-75%0 and 50% H-50%0. Nevertheless, the bicontinuous bigel structure was produced by the bigel samples with a 75% hydrogel ratio. Similar results were reported for the konjac glucomannan–gelatin bigel samples.^39^ These results suggested that the primary determinant of bigel microstructure development is the hydrogel/Oleogel ratio.^39^

**Figure 2:**
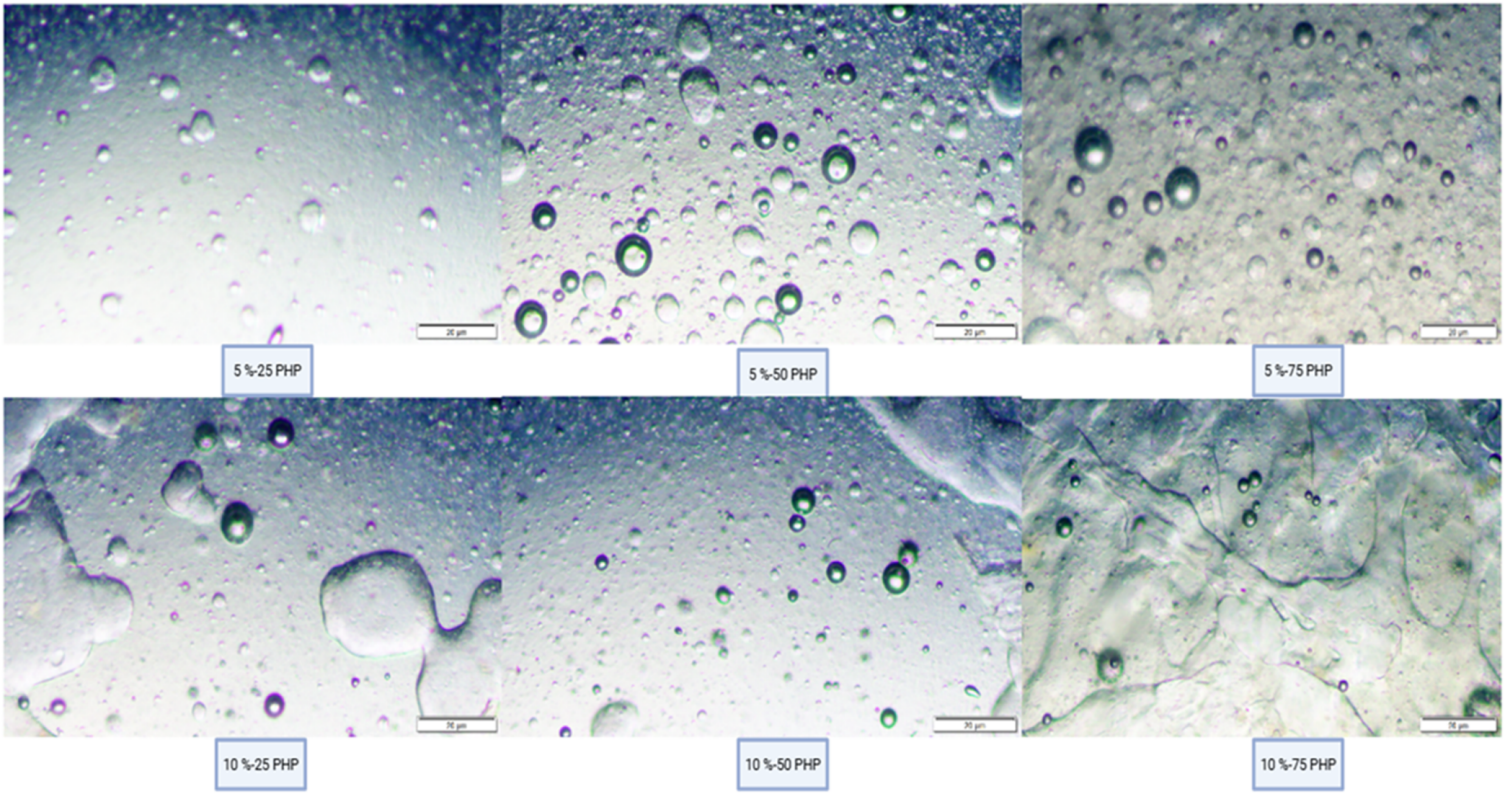
Microstructural Properties of Bigels (5%-25 PHP: containing 5% psyllium husk seed powder with 25:75 psyllium husk seed powder hydrogel to beeswax Oleogel ratio, 5%-50 PHP: containing 5% psyllium husk seed powder with 50:50 psyllium husk seed powder hydrogel to beeswax Oleogel ratio, 5%-75 PHP: containing 5% psyllium husk seed powder with 75:25 psyllium husk seed powder hydrogel to beeswax Oleogel ratio, 10%-25 PHP: containing 10% psyllium husk seed powder with 25:75 to beeswax Oleogel ratio, 10%-50 PHP: containing 10% psyllium husk seed powder with 50:50 psyllium husk seed powder hydrogel to beeswax Oleogel ratio 10%-75 PHP: containing 10% psyllium husk seed powder with 75:25 psyllium husk seed powder hydrogel to beeswax Oleogel ratio).

Bicontinuous structures were created by using 5 and 10% bigel at a hydrogel/Oleogel volume ratio of 75:25. Furthermore, the hydrogel and Oleogel phases did not separate from one another; instead, they interacted synergistically because one was unevenly distributed over the other. The findings showed that these bigels lacked a transparent barrier between the hydrogel and Oleogel phases and exhibited a bicontinuous emulsion structure.^40^ Similar results were reported by Xie et al.^36^

### 3.2 FTIR

FTIR was used to study the intermolecular forces in the big els. Fourier transform infrared spectroscopy (FTIR) analysis was used to determine the distinctive peaks of each bigel component. The FTIR spectra of the Oleogel, hydrogels, and bigels are given in Figure 3. No new peaks were identified in the bigel samples with different fractions of Oleogel, demonstrating that the combination of PHP hydrogel and BW Oleogel was physical, with no chemical interaction. Similar results were reported by ref 41,42

**Figure 3:**
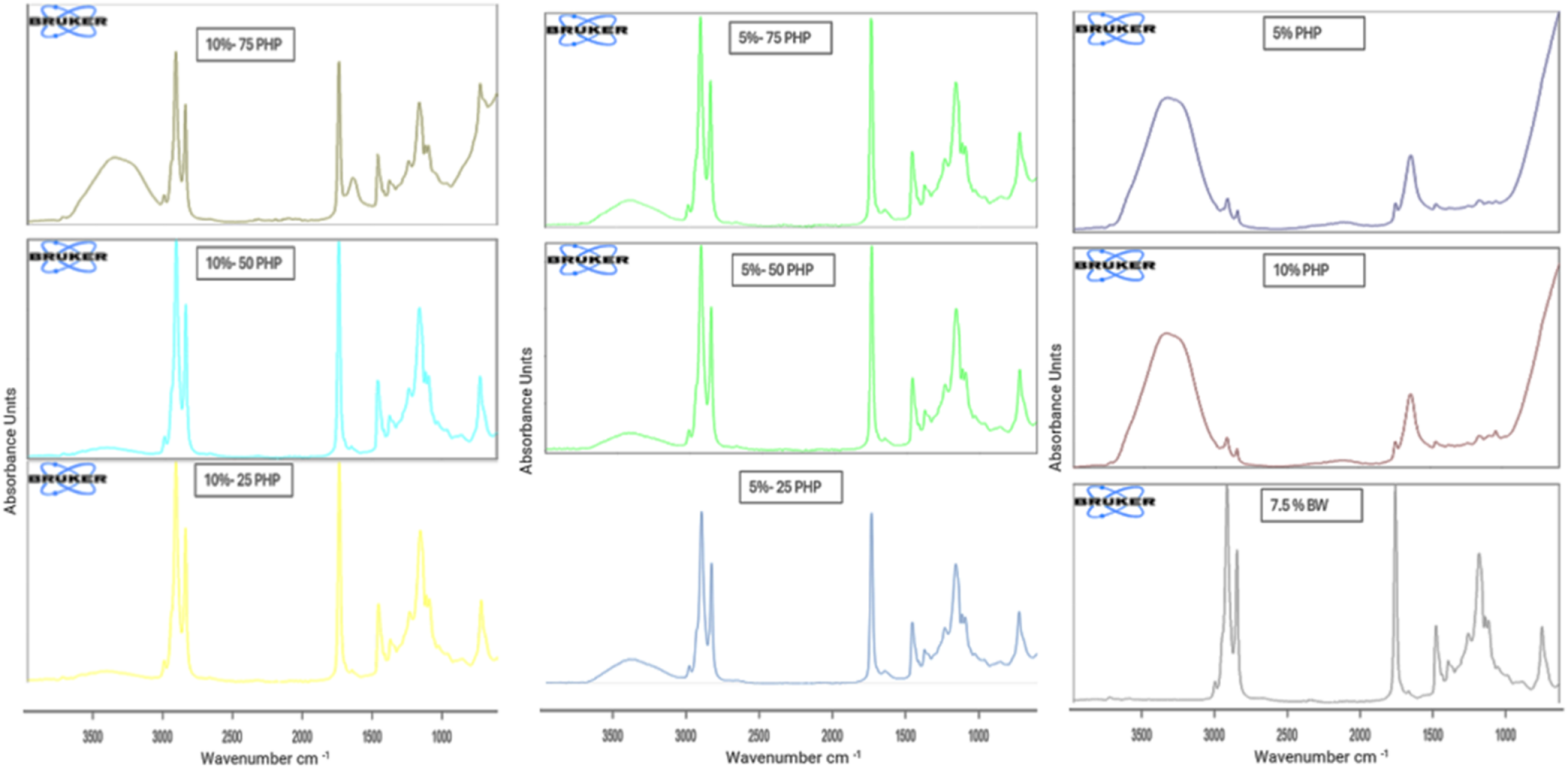
FTIR of Oleogel, hydrogels, and bigel samples (7.5% BW: containing 7.5% beeswax Oleogel, 10% PHP: containing 10% psyllium husk seed powder, 5% PHP: containing 5% psyllium husk seed powder, 5%-25 PHP: containing 5% psyllium husk seed powder with 25:75 psyllium husk seed powder hydrogel to beeswax Oleogel ratio, 5%-50 PHP: containing 5% psyllium husk seed powder with 50:50 psyllium husk seed powder hydrogel to beeswax Oleogel ratio, 5%-75 PHP: containing 5% psyllium husk seed powder with 75:25 psyllium husk seed powder hydrogel to beeswax Oleogel ratio, 10%-25 PHP: containing 10% psyllium husk seed powder with 25:75 psyllium husk seed powder hydrogel to beeswax Oleogel ratio, 10%-50 PHP: containing 10% psyllium husk seed powder with 50:50 psyllium husk seed powder hydrogel to beeswax Oleogel ratio 10%-75 PHP: containing 10% psyllium husk seed powder with 75:25 psyllium husk seed powder hydrogel to beeswax Oleogel ratio).

The bigel samples showed a broad band at 3700–3000 cm–1, corresponding to the O–H stretching vibrations from the fatty acyl molecules in Oleogel and the water molecules in PHP hydrogel.^41,43^ The strength of this peak rapidly dropped as the Oleogel/hydrogel ratio increased, indicating a decline in the hydrogen bonding in bigels. Similar results were reported for the peaks at 2900, 2800, and 1460 cm–1, which indicate C–H or C=H stretching vibrations from saturated and unsaturated fatty acyl chains in the oil phase.^42,44^ The OG detected beeswax bands at 2929 and 2851 cm–1, indicating asymmetric and symmetric stretching of aliphatic hydrocarbons.^45,46^ Increasing the hydrogel/Oleogel ratio lowered the absorption signals at 1740 and 1160 cm–1, which reflect C=O stretching vibrations in the oil phase.^42^ Absorption bands at 1746 and 1160 cm–1 indicate the presence of carboxyl groups in fatty acids and esters.^46,47^

### 3.3 Rheological Properties of Oleogel, Hydrogels, and Bigels

The storage and loss modulus values of the bigel, hydrogel, and Oleogel samples are displayed in Figure 4. Across the frequency value, there was no crossover point, and the *G*′ value was consistently greater than the *G*″ value in every sample. This is the primary attribute that defines gel formations. These results indicate that the samples showed a semisolid structure.^48,49^

**Figure 4:**
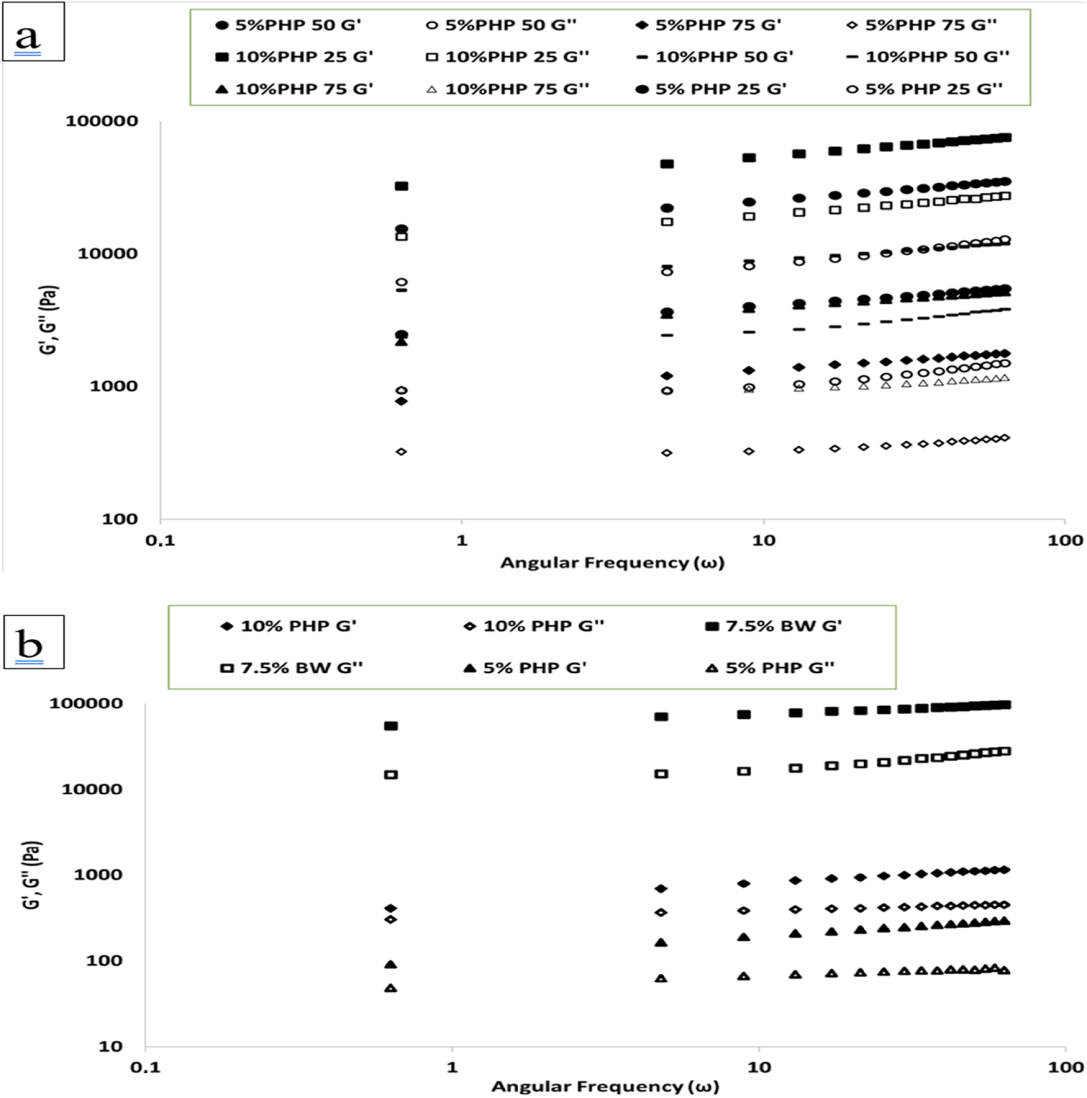
(a, b). Dynamic rheological properties of the Oleogel. Hydrogel and bigel samples (7.5% BW: containing 7.5% beeswax Oleogel, 10% PHP: containing 10% psyllium husk seed powder, 5% PHP: containing 5% psyllium husk seed powder, 5%-25 PHP: containing 5% psyllium husk seed powder with 25:75 psyllium husk seed powder hydrogel to beeswax Oleogel ratio, 5%-50 PHP: containing 5% psyllium husk seed powder with 50:50 psyllium husk seed powder hydrogel to beeswax Oleogel ratio, 5%-75 PHP: containing 5% psyllium husk seed powder with 75:25 psyllium husk seed powder hydrogel to beeswax Oleogel ratio, 10%-25 PHP: containing 10% psyllium husk seed powder with 25:75 psyllium husk seed powder hydrogel to beeswax Oleogel ratio, 10%-50 PHP: containing 10% psyllium husk seed powder with 50:50 psyllium husk seed powder hydrogel to beeswax Oleogel ratio 10%-75 PHP: containing 10% psyllium husk seed powder with 75:25 psyllium husk seed powder hydrogel to beeswax Oleogel ratio).

Figure 4 demonstrates that the frequency increase did not change the hydrogel, Oleogel, and bigel samples. The structure of the bigel, hydrogel, and Oleogel samples was independent of frequency. This indicates the formation of a firm gel structure.

Figure 4B showed that *G*′ rose as the concentration of PHP hydrogel increased, with Oleogel having a greater G ′ value than the hydrogel samples. All bigel samples exhibited solid properties closer to pure Oleogel than hydrogel. Oleogel’s stiffer structure may have played a significant role in determining the rheological signature of bigel systems..^50^ Figure 4A demonstrated that the *G*′ values of bigels rise with hydrogel concentration and Oleogel ratio. 10–25 PHP showed higher *G*′ values than all of the bigel samples. 5–75 PHP showed lower *G*′ values than the other bigel samples. An increase in the Oleogel ratio in bigel samples led to an increase in *G*′ values at 5% and 10% PHP concentrations. 10% PHP bigel samples showed higher *G*′ values than the 5% PHP bigel samples across all hydrogel/Oleogel ratios. An increase in hydrogel concentration led to a rise in bigel strength. Bigels demonstrated enhanced solid characteristics (*G*′) compared with individual gels, implying a synergistic effect among phases. Several variables may have contributed to this improvement.^50^

The enhanced viscoelasticity (*G*′ and *G*″ during frequency sweep) of bigels is believed to be caused by the discrete phase, which may operate as a “interacting filler” (hydrogel discrete phase for bigels with 50 and 25% hydrogel).^48,49^ Pure hydrogel had lower *G*′ values, whereas pure Oleogel had higher *G*′ values than bigels. Hydrogel discrete phase (5–25, 5–50 and 10–25, 10–50% PHP) with an increase in Oleogel ratio demonstrated synergistic impact and enhanced *G*′ values of bigels. The dispersed phase will operate as a filler and play an essential role in improving the strength of the bigels.^36,51^ *G*′ value increased as the Oleogel ratio increased from 25 to 50 in the bigel at 5 and 10% bigel concentrations (Figure 4A). This can be attributed to the synergistic effect between the hydrogel and Oleogel structures.

Results from studies on the dynamic rheological characteristics of bigels, oleogels, and hydrogels were compared by using the power law model. The dynamic rheological characteristics of the materials can be accurately simulated by this model, as demonstrated by Table 2 (*R* ^2^ > 0.99). *K*′ values larger than *K*″ were found in all samples, suggesting viscoelastic solid behavior. The results for *n*′ and *n*″ also indicate that the samples are viscoelastic solids. The sample’s solid quality becomes increasingly visible as these values approach zero. An increase in the Oleogel ratio led to an increase in *K*’s value from 900.29 to 16578.37 for 5% PHP and from 2594.94 to 35654.37 for 10% PHP bigel samples.

**Table 2:** Dynamic Rheological Properties of Bigels^a^

sample	K′(Pa.s*^n′^*)	*n*′	*R* ^2^	K″(Pa.s^n′′^)	*n*″	*R* ^2^
7.5% BW	57599.08 ± 11832.17a	0.125 ± 0.007	0.99	11462 ± 1737.34a	0.205 ± 0.049	0.925 ± 0.035
5% PHP	112.40 ± 20.47d	0.234 ± 0.016	0.99	52.69 ± 5.258c	0.107 ± 0.007	0.98
10% PHP	896.27 ± 54.23 cd	0.2201 ± 0.028	0.99	317.2 ± 55.74c	0.085 ± 0.001	0.99 ±
5%-25 PHP	16578.37 ± 1971.80c	0.18 ± 0	0.99	5457.94 ± 1204.853b	0.198 ± 0.0007	0.976 ± 0.008
5%- 50 PHP	2748.83 ± 45.022 cd	0.165 ± 0.0014	0.99	770.07 ± 6.790c	0.147 ± 0.001	0.907 ± 0.0106
5% - 75 PHP	900.29 ± 290.78 cd	0.165 ± 0.0012	0.99	292.83 ± 93.891c	0.065 ± 0.0134	0.852 ± 0.0178
10%- 25 PHP	35654.37 ± 1.244b	0.185 ± 0.007	0.99	13682.16 ± 162.563a	0.165 ± 0.007	0.99 ±
10% - 50 PHP	6040.01 ± 1221.43 cd	0.166 ± 0	0.99	1986.22 ± 408.594c	0.143 ± 0.0007	0.926 ± 0.008
10% - 75 PHP	2594.94 ± 25.187 cd	0.167 ± 0.004	0.99	887.45 ± 43.196c	0.054 ± 0.002	0.83 ± 0.0282

a a–d: Different superscript letters indicate significant differences between samples in the same column (*p* < 0.05).

Despite a numerical difference between the samples, the influence of the hydrogel/Oleogel ratio on the *K*′ value of the samples was not statistically significant, except for the 10–25 PHP% sample, and *K*″ values of the samples were not statistically significant, except the 5–25 and 10–25 PHP% samples. The BW had a greater *K* value than other hydrogel and bigel samples (*p* < 0.05). The 5–25 PHP% and 10–25 PHP% samples had higher *K*′ values compared to the 5 and 10% PHP samples (*p* < 0.05). This finding might be explained by the synergistic effects of Oleogel and hydrogel samples. The results were consistent with the *G*′ and *G*″ values of the gel samples. The *K*″ values of the 10–25 PHP% and BW were not statistically significant.

Figure 5 showed that the storage modulus (*G*′) of Oleogels, hydrogels, and bigels at temperatures ranging from 20 to 80 °C. The increase in hydrogel ratio increased *G*′ (Figure 5). The hydrogels have *G* values higher than those of the Oleogel. Incorporating hydrogels into the Oleogel increased bigels’ elastic properties at temperatures below 60 °C. Similar results were reported by Habibi et al.^5^ During the early heating phase, a sharp decrease in the storage modulus following a critical temperature of around 40 °C was attributed to the system’s instability.^52^ Bigels with 5–75 PHP% % and 10–75 PHP% % offered greater temperature stability and a more gradual abrupt transition. The temperature, melting of BW crystals, and temperature resistance of PHP hydrogels all have a significant effect on the *G*′ of the BGs. Similar results were reported by Barroso et al.^53^ for potato starch hydrogel and glycerol monostearate-based bigels.

**Figure 5:**
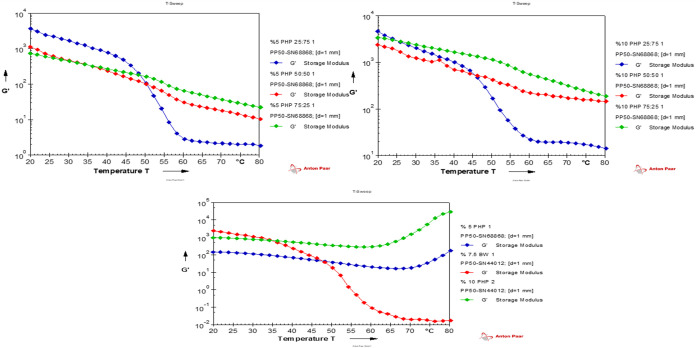
Temperature sweep test of Oleogel, hydrogel, and bigel samples, *n* = 1. (7.5% BW: containing 7.5% beeswax Oleogel, 10% PHP: containing 10% psyllium husk seed powder, 5% PHP: containing 5% psyllium husk seed powder, 5%-25 PHP: containing 5% psyllium husk seed powder with 25:75 psyllium husk seed powder hydrogel to beeswax Oleogel ratio, 5%-50 PHP: containing 5% psyllium husk seed powder with 50:50 psyllium husk seed powder hydrogel to beeswax Oleogel ratio, 5%-75 PHP: containing 5% psyllium husk seed powder with 75:25 psyllium husk seed powder hydrogel to beeswax Oleogel ratio, 10%-25 PHP: containing 10% psyllium husk seed powder with 25:75 psyllium husk seed powder hydrogel to beeswax Oleogel ratio, 10%-50 PHP: containing 10% psyllium husk seed powder with 50:50 psyllium husk seed powder hydrogel to beeswax Oleogel ratio 10%-75 PHP: containing 10% psyllium husk seed powder with 75:25 psyllium husk seed powder hydrogel to beeswax Oleogel ratio).

### 3.4 Texture Profile Analysis of Hydrogels, Oleogel, and Bigels

Samples’ resistance to deformation is correlated with their hardness. Springiness describes a food’s capacity to regain its original shape after deformation.^36^ The textural properties of pure Oleogel, hydrogels, and bigels with different Oleogel/hydrogel ratios are presented in Table 3, including hardness, cohesiveness, springiness, chewiness, and gumminess.

**Table 3:** Textural Properties of Bigel Samples^a^

	hardness (g)	springiness	cohesiveness	gumminess	chewiness
7.5% BW	47.435 ± 7.9c	0.904 ± 0.068a	0.701 ± 0.043bc	33.046 ± 3.525d	29.913 ± 4.353d
5% PHP	202.149 ± 45.694bc	0.947 ± 0.001a	0.826 ± 0.044ab	166.043 ± 28.889c	157.19 ± 27.105c
10% PHP	1018.4 ± 193.20a	0.711 ± 0.072b	0.655 ± 0.02c	664.662 ± 104.3a	467.9 ± 31.7a
5%-25 PHP	48.9425 ± 11.9c	0.9515 ± 0.007a	0.803 ± 0.05ab	39.593 ± 12.15 cd	37.7265 ± 11.87 cd
5%- 50 PHP	71.154 ± 6.86bc	0.948 ± 0.003a	0.786 ± 0.038abc	55.741 ± 2.713 cd	52.844 ± 2.545 cd
5% - 75 PHP	71.452 ± 3.021c	0.941 ± 0.007a	0.786 ± 0.074abc	56.248 ± 6.977 cd	52.986 ± 6.927 cd
10%- 25 PHP	91.231 ± 15.418c	0.953 ± 0.003a	0.789 ± 0.049ab	71.69 ± 10.65 cd	68.339 ± 10.387 cd
10%- 50 PHP	142.931 ± 6.002c	0.953 ± 0.008a	0.797 ± 0.045ab	113.744 ± 4.757 cd	108.364 ± 5.296 cd
10%- 75 PHP	359.943 ± 17.88b	0.964 ± 0.001a	0.844 ± 0.007a	303.694 ± 13.743b	292.677 ± 13.455b

a a–d: Different superscript letters indicate significant differences between samples in the same column (*p* < 0.05).

The hydrogels and bigels showed higher hardness values than the Oleogel. However, an increased hydrogel ratio in bigels led to increased hardness values. The hardest material was a hydrogel with 10% PHP, while the softest was 7.5% BW Oleogel. Oleogel produced a creamier gel, but the hydrogels were brittle. Also, the increase in the hydrogel ratio in bigel led to increased gumminess and chewiness. The discrete phase of bigels will operate as a “active filler,” allowing for a superior gel structure. This will increase the hardness.^48,54^

Pure Oleogel and 10% PHP had the lowest springiness value among gel samples, whereas 10%-75 PHP bigel samples had the highest. The lowest springiness values indicate poor shape recovery following deformation. Similar results were reported by Xie et al.^36^ The bigel samples showed higher springiness values than those of the pure hydrogels and Oleogel. This behavior was in line with the rheological findings, which is explained by the concept that the bigels’ dispersed phase will act as a filler to increase their strength.^51^

The percentage of hydrogel to Oleogel consistently affected taste-related aspects of the bigel, including cohesiveness, gumminess, and chewiness (*p* < 0.05). Similar results were reported by Jiang et al.^41^ Generally, the BW Oleogel to PHP hydrogel ratio might be adjusted to control the texture of bigels.

### 3.5 Stability of Bigels

Figure 6 displays the bigel samples’ accelerated percolation rate following centrifugation.

**Figure 6:**
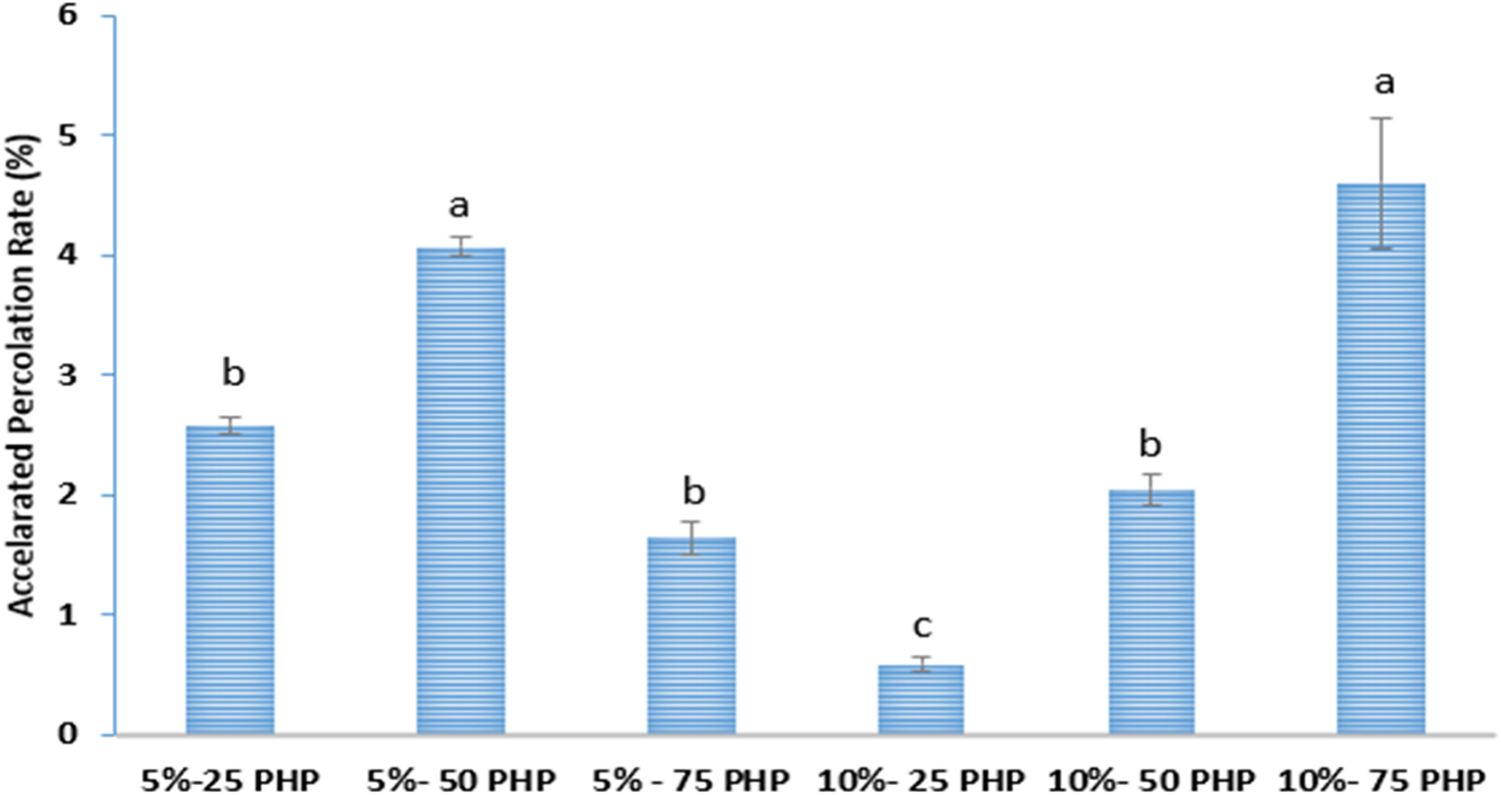
Stability of the bigel samples after centrifugation. Different lowercase letters highlight significant differences observed between groups (*n* = 2, Mean ± SD,*p*< 0.05).(5%-25 PHP: containing 5% psyllium husk seed powder with 25:75 psyllium husk seed powder hydrogel to beeswax Oleogel ratio, 5%-50 PHP: containing 5% psyllium husk seed powder with 50:50 psyllium husk seed powder hydrogel to beeswax Oleogel ratio, 5%-75 PHP: containing 5% psyllium husk seed powder with 75:25 psyllium husk seed powder hydrogel to beeswax Oleogel ratio, 10%-25 PHP: containing 10% psyllium husk seed powder with 25:75 psyllium husk seed powder hydrogel to beeswax Oleogel ratio, 10%-50 PHP: containing 10% psyllium husk seed powder with 50:50 psyllium husk seed powder hydrogel to beeswax Oleogel ratio 10%-75 PHP: containing 10% psyllium husk seed powder with 75:25 psyllium husk seed powder hydrogel to beeswax Oleogel ratio).

An increase in hydrogel concentration led to an increase in the accelerated percolation rate of bigels, except for 5%-75 PHP. When the hydrogel ratio changed from 25 to 50, the accelerated percolation rate increased from 2.58 ± 0.06 to 4.07 ± 0.07. Then, the hydrogel ratio of 50 to 75% accelerated percolation, decreasing the rate to 1.64 ± 0.13. When the hydrogen ratio increased to 5%-75 PHP, PHP was incorporated into the BW crystal structure by hydrogen bonding, creating a continuous network structure that improved the gel network’s ability to physically regulate liquid oil.^17,55^ Moreover, Singh et al. (2014)^56^ found a strong correlation between the Oleogel/hydrogel ratios and the physical stability of bigels.

The accelerated percolation rate of bigels rose from 0.584 ± 0.06 to 4.60 ± 0 54 when the PHP ratio rose from 25 to 75%. This might happen as a result of the PHP-BW continuous network structure being disrupted, the accelerated percolation rate increasing, and needle-like, plate-like, and spherulite crystals were created when the PHP was implanted in the BW crystal structure, achieving saturation.^17,57^

With the lowest accelerated percolation rate, the bigel (10%–25 PHP) was the most stable sample and maintained the most liquid. As a result, 10%-25 PHP was used to replace palm oil in cakes.

### 3.6 Visual Appearance of Dough’s Surface, Cake’s Surface, and Cross sections

Figure 7 shows the visual appearance of dough’s and cake’s surfaces, and cross-sectional views of cakes produced with PHP bigels and palm oil (control). The increase in hydrogel ratio from 25 to 75% resulted in a more porous cake sample. The Control sample was found to have a compact structure, less pore space, and no network development that would have allowed for the incorporation and retention of more gas. As seen in doughs, integrity was not achieved in the cookie dough obtained from bigels in which 5%-50 PHP, 5%-75 PHP and 10%-50 PHP, 10%-75 PHP hydrogels were used, and there was a slip on the top of the cakes after baking. Cake samples created with palm oil, 5%-25 PHP, and 10%-25 PHP had a crumb structure with fine air cells that were evenly distributed, but cakes made with 10%-50 PHP and 10%-75 PHP had comparatively big pores with gas cells. This might be due to extra air bubbles being added to the batter created using these samples during the mixing process.^33^ Higher BW ratio (75%) in bigel samples showed a homogeneous structure. The results agreed with those reported by Oh et al,^58^ cakes prepared with three different oleogels as a replacement for shortening. Oh et al^58^ found that the cake samples prepared with BW and shortening showed more homogeneously distributed fine air cells.

**Figure 7:**
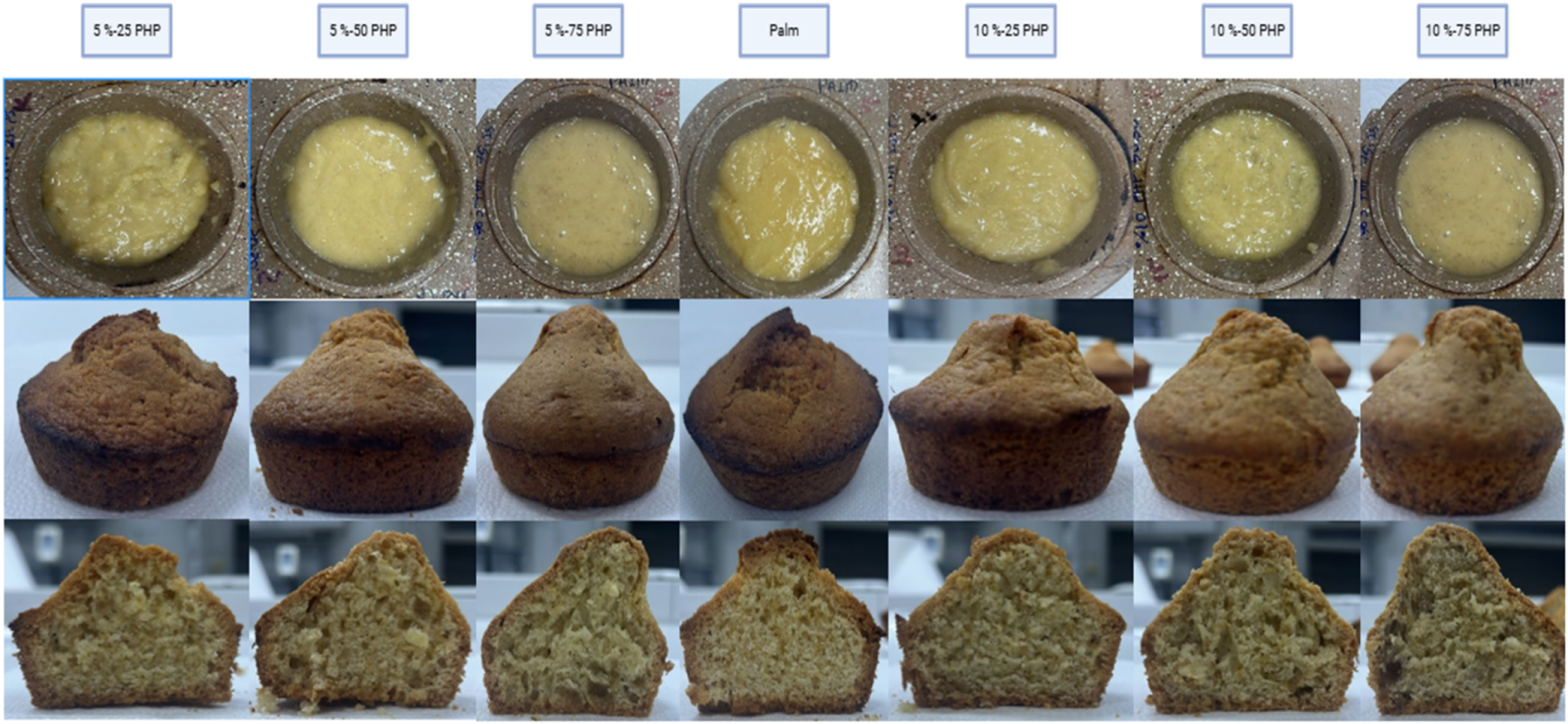
Visual appearance of dough surface, cake surface, and cross sections (palm: containing palm oil in cake, 5%-25 PHP: containing 5% psyllium husk seed powder with 25:75 psyllium husk seed powder hydrogel to beeswax Oleogel ratio, 5%-50 PHP: containing 5% psyllium husk seed powder with 50:50 psyllium husk seed powder hydrogel to beeswax Oleogel ratio, 5%-75 PHP: containing 5% psyllium husk seed powder with 75:25 psyllium husk seed powder hydrogel to beeswax Oleogel ratio, 10%-25 PHP: containing 10% psyllium husk seed powder with 25:75 psyllium husk seed powder hydrogel to beeswax Oleogel ratio, 10%-50 PHP: containing 10% psyllium husk seed powder with 50:50 psyllium husk seed powder hydrogel to beeswax Oleogel ratio 10%-75 PHP: containing 10% psyllium husk seed powder with 75:25 psyllium husk seed powder hydrogel to beeswax Oleogel ratio).

The cakes most similar to the control cakes in the cross-sectional view were those prepared from 5%-25 PHP and 10%-25 PHP bigels.

### 3.7 Steady Shear Rheological Properties of Cake Dough

Figure 8 displays the flow behavior curves for cake batters (CB) made with different bigels and control (palm oil) and shows how increasing the shear rate resulted in a decreasing trend for shear stress. This relationship between shear stress and shear rate indicates shear thinning behavior, consistent with the results of earlier research.^33,59^

**Figure 8:**
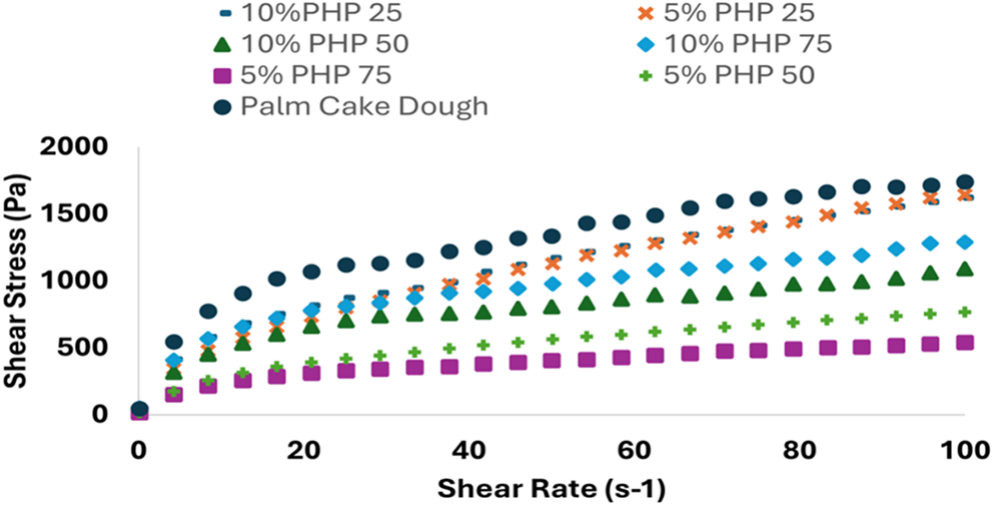
Steady shear rheological properties of the cake batters (palm cake dough: containing palm oil in cake dough, 5%-25 PHP: containing 5% psyllium husk seed powder with 25:75 psyllium husk seed powder hydrogel to beeswax Oleogel ratio, 5%-50 PHP: containing 5% psyllium husk seed powder with 50:50 psyllium husk seed powder hydrogel to beeswax Oleogel ratio, 5%-75 PHP: containing 5% psyllium husk seed powder with 75:25 psyllium husk seed powder hydrogel to Beeswax Oleogel ratio, 10%-25 PHP: containing 10% psyllium husk seed powder with 25:75 psyllium husk seed powder hydrogel to beeswax Oleogel ratio, 10%-50 PHP: containing 10% psyllium husk seed powder with 50:50 psyllium husk seed powder hydrogel to beeswax Oleogel ratio 10%-75 PHP: containing 10% psyllium husk seed powder with 75:25 psyllium husk seed powder hydrogel to beeswax Oleogel ratio).

The consistency index (*K*) and flow behavior index (*n*) of the cake batters, as determined by the power law model (*R* ^2^ > 0.95), are shown in Table 4. The consistency coefficient (*K*) and flow behavior index (*n*) values varied from 95.66 to 448.48 Pa.s*^n^* and from 0.18 to 0.52. The results showed that the formulation’s different hydrogel/Oleogel ratios significantly impacted the rheological characteristics. The 10%-75 PHP sample had the most excellent *K* value, whereas CB-5%-50 and 5%-75 PHP had lower values.

**Table 4:** Steady Shear Rheological Parameters of Cake Batters^a^

sample	*K* (Pa.s*^n^*)	*n*	*R* ^2^
palm cake dough	357.85 ± 50.54a	0.35 ± 0.07	0.99
5%-25 PHP	150.65 ± 14.38bc	0.52 ± 0.04	0.99
5%- 50 PHP	99.89 ± 2.33c	0.44 ± 0.036	0.99
5% - 75 PHP	95.66 ± 6.44c	0.37 ± 0.03	0.99
10%- 25 PHP	208.96 ± 12.26b	0.44 ± 0.01	0.99
10% - 50 PHP	221.13 ± 7.09b	0.34 ± 0.03	0.99
10% - 75 PHP	408.48 ± 28.96a	0.18 ± 0.02	0.95

a a–c: Different superscript letters indicate significant differences between samples in the same column (*p* < 0.05).

### 3.8 Texture Profile Analysis of Cakes

The texture profile parameters of the cakes are given in Table 5. The presence of the gelled aqueous phase gives bigel dough a distinct texture. The results revealed by hydrogelators in bigels prevent the flour particles from getting hydrated by blocking the movement of water molecules in the system. Conversely, less plasticization of the dough produced harder dough than expected.^60^ Control cakes showed the highest hardness value (1636.16). The hardness values of the 5% PHP-25 and 10% PHP-25 cake samples were found closest to the hardness values of the control cakes. However, there were no significant differences between the control cake and bigel cake samples, except for the 10%-50 PHP.

**Table 5:** Textural Properties of Cake Samples^a^

	cakes’ texture profile				
	hardness (g)	springiness	cohesiveness	gumminess	chewiness
control	1636.16 ± 118.66a	0.861 ± 0.061a	0.4465 ± 0.068ab	2286.746 ± 235.19a	1883.34 ± 319.7a
5%-25 PHP	1505.6 ± 382.98ab	0.802 ± 0.095a	0.25 ± 0.059b	503.16 ± 82.13b	414.27 ± 59.55b
5%- 50 PHP	946.017 ± 152.23ab	0.9065 ± 0.026a	0.559 ± 0.037ab	531.40 ± 108.74b	502.89 ± 60.19b
5% - 75 PHP	1095.348 ± 301.9ab	0.947 ± 0.027a	0.527 ± 0.085ab	560.38 ± 60.01b	531.61 ± 67.78b
10%- 25 PHP	1428.78 ± 226.83ab	0.851 ± 0.095a	0.385 ± 0.066ab	542.88 ± 7.40b	491.91 ± 66.31b
10%- 50 PHP	813.40 ± 103.29b	0.873 ± 0.041a	0.517 ± 0.108ab	413.166 ± 40.929b	361.57 ± 51.22b
10%- 75 PHP	1147.79 ± 124.36ab	0.965 ± 0.072a	0.638 ± 0.115a	520.01 ± 58.87b	496.61 ± 68.19b

a a–b: Different superscript letters indicate significant differences between samples in the same column (*p* < 0.05).

Also, there were no significant differences between the control and bigel cake samples regarding springiness and cohesiveness values. The control cakes’ gumminess and chewiness values were higher than the bigel cake samples (*p* < 0.05). Although the bigel samples showed significant differences in gumminess and chewiness values, bigels could be used instead of palm oil to replace cakes’ inherent breakability.

### 3.9 Color Properties of the Cakes

The crumb and crust color properties of cakes obtained with bigels and palm oil prepared using PHP and BW in different concentrations and ratios are presented in Table 6. The crust *L* value of the control samples prepared using palm oil was determined as 44.6 ± 0.68b, the *a* value was 13.80 ± 1.33a, and the *b* value was 23,50 ± 1,00b. The *L* values of cakes were in the range of 44.6 −56.29. The higher this value, the lighter the color. The 5%-25 PHP cake sample showed the highest *L* value (56.29). The hydrogel/Oleogel ratio did not lead to a significant change in the *L* value. The crust of the control cakes had a lower *L* value than that of the bigel samples. According to some reports, cakes made with shortening, have a darker crust color. Similar results were reported by ref 61,62 ^62^ cakes seemed darker than Oleogel cakes.

**Table 6:** Color Properties of the Crust and Crumb of Cakes^a^

		crust		
	L*	a*	b*	*ΔE*
control	44.6 ± 0.68b	13.80 ± 1.33a	23.50 ± 1.00b	-
5%-25 PHP	56.29 ± 1.32a	10.33 ± 0.89b	25.89 ± 0.52ab	12.38 ± 1.38a
5%- 50 PHP	55.88 ± 1.57a	11.99 ± 0.49ab	28.76 ± 1.57a	12.54 ± 2.05a
5% - 75 PHP	55.86 ± 2.11a	10.27 ± 0.92b	25.90 ± 2.20ab	12.06 ± 2.73a
10%- 25 PHP	52.99 ± 0.97a	11.66 ± 0.60ab	24.73 ± 0.56ab	8.70 ± 1.04a
10%- 50 PHP	55.44 ± 0.51a	9.92 ± 0.64b	26.83 ± 1.96ab	12.05 ± 0.18a
10%- 75 PHP	54.4 ± 1.31a	10.28 ± 0.57b	23.64 ± 1.66b	10.43 ± 1.50a

crumb				
	L*	a*	b*	*ΔE*
control	67.38 ± 0.71ab	1.88 ± 0.24a	26.59 ± 0.69a	-
5%-25 PHP	61.29 ± 1.21c	1.60 ± 0.09ab	22.40 ± 1.11bc	7.41 ± 1.52ab
5%- 50 PHP	64.72 ± 1.74abc	1.65 ± 0.17ab	23.25 ± 1.00b	4.36 ± 1.69b
5% - 75 PHP	67.75 ± 2.39a	–0.74 ± 0.11e	22.16 ± 0.87bc	5.56 ± 0.34b
10%- 25 PHP	63.67 ± 1.32bc	1.32 ± 0.12bc	22.52 ± 0.60bc	5.55 ± 1.34b
10%- 50 PHP	65.71 ± 0.61ab	0.38 ± 0.09d	22.63 ± 0.59b	4.57 ± 0.65b
10%- 75 PHP	61.09 ± 0.84c	0.97 ± 0.04c	19.89 ± 1.58c	9.25 ± 1.71a

a a–e: Different superscript letters indicate significant differences between samples in the same column (*p* < 0.05).

The *b* values of the cakes varied between 23.50 and 28.76. The 5%-50 PHP cake samples showed the highest *b* value (28.76). The highest *b* value indicated that it was closer to the yellow color. There were no significant differences between the *b* value of the crust of the control cakes and bigel cakes, except 5%-50 PHP. The b values decreased for the bigel cakes, indicating that shortening replacement with oleogels resulted in lower yellowness values, consistent with the pictures in Figure 7.

A total color difference (Δ*E*) of >3 indicates that the difference is apparent to the naked eye. Δ*E* values of the crust bigel cake samples were between 8.70 and 12.54. 10%–25% of the PHP bigel cake samples were similar to the control cake crumb. Δ*E* values of the crumb bigel cake samples were between 4.36 and 9.25. 5%–50% of the PHP bigel cake samples were similar to the control cake crumb. Bigels cakes with 5% PHP-50 showed the least color change based on Δ*E* analysis. The highest color change was observed in bigels prepared with a 10% PHP-75 ratio for crumb cakes. Several factors, including cake composition, oil or replacement, and baking conditions, affect color values. Customer acceptability of the product is the primary determinant. From this point of view, it has been shown that cakes made with oleogels that contain a lot of unsaturated fat may be used in the baking industry and have color features comparable to cakes made with shortening.^61^

## 4 Conclusions

This study examines the use of PHP in manufacturing bigels and its application as a substitute for palm oil in cakes; bigels exhibited solid qualities that were viscoelastic. The *G*′ values of the 10 to 25% PHP and 5–25% bigel samples were greater than those of the pure hydrogel. Compared with other bigel samples, these samples also had greater *K*′ values. The HG/OG ratio did not significantly impact the *K*′ values, except for 10%–25 PHP. The temperature sweep test also showed that a greater PHP concentration or ratio enhanced thermal stability. Bigel samples were used in cake making instead of palm oil in the formulation. Cake samples created with palm oil, 5%-25 PHP, and 10%-25 PHP had a crumb structure with fine air cells evenly distributed. The cakes’ visual appearance and textural and color properties were evaluated by comparing them to control palm cakes. The hardness values of 10%-25 PHP and 5%-25 PHP cakes showed no significant change with the control palm cakes (p 0.05). Furthermore, based on the Δ*E* value, 10%-25 PHP cakes had the least color change in the crust color of the cake. After baking, the intended fat functionality was preserved, and bigel cakes exhibited texture, visual, and color characteristics comparable to those of palm cakes. The 10%-25 PHP cake sample would be a better choice as it could be considered an alternative to palm oil in cakes regarding visual appearance, textural properties, and color properties. 10%-25 PHP-based bigel can be successfully used as a palm oil substitute for cakes. Overall, bigels comprised a suitable alternative to palm oil in the production of cakes. Further research might focus on encapsulating antioxidant compounds in PHP modules and evaluating their potential health benefits to understand PHP comprehensively.

## Supplementary Material



## Data Availability

The data that support the findings of this study are available from the corresponding author upon reasonable request.

## Notes

FTIR Raw Data (XLSX)
